# Using cAMP Sensors to Study Cardiac Nanodomains

**DOI:** 10.3390/jcdd5010017

**Published:** 2018-03-13

**Authors:** Katharina Schleicher, Manuela Zaccolo

**Affiliations:** Department of Physiology, Anatomy and Genetics, University of Oxford, Sherrington Building, South Parks Road, Oxford OX1 3PT, UK; katharina.schleicher@dpag.ox.ac.uk

**Keywords:** 3′,5′-cyclic adenosine monophosphate, protein kinase A, fluorescence resonance energy transfer, real-time imaging, compartmentalisation, signalling, cardiac biology, phosphodiesterases, A kinase anchoring proteins

## Abstract

3′,5′-cyclic adenosine monophosphate (cAMP) signalling plays a major role in the cardiac myocyte response to extracellular stimulation by hormones and neurotransmitters. In recent years, evidence has accumulated demonstrating that the cAMP response to different extracellular agonists is not uniform: depending on the stimulus, cAMP signals of different amplitudes and kinetics are generated in different subcellular compartments, eliciting defined physiological effects. In this review, we focus on how real-time imaging using fluorescence resonance energy transfer (FRET)-based reporters has provided mechanistic insight into the compartmentalisation of the cAMP signalling pathway and allowed for the precise definition of the regulation and function of subcellular cAMP nanodomains.

## 1. Introduction

Cyclic nucleotides, such as 3′,5′-adenosine monophosphate (cAMP), are small molecules used by cells to propagate extracellular information inside the cell and are referred to as second messengers. cAMP is generated by intracellular adenylyl cyclases in response to a first, extracellular message that activates a transmembrane G-protein coupled receptor (GPCR). This second messenger system is used in many types of cells, from prokaryotes to human neurons, and in each cell type its synthesis can be triggered by different extracellular stimuli with different functional outcomes. It is thus imperative to understand how a universal signal like cAMP can be versatile enough to generate cellular effects that are specific to each individual stimulus. This question demands sensitive quantification of the second messenger and accurate comparison of cAMP signal amplitude, dynamics, and subcellular location in response to different extracellular cues.

In the cardiovascular system, the first message that activates the cAMP pathway is provided by a variety of biochemically diverse molecules. These include neurotransmitters, such as adrenaline and noradrenaline; peptide hormones, such as glucagon; and lipid compounds, such as prostaglandins. Each molecule binds to a distinct GPCR, coupling to different kinds of G-proteins with the ability to activate or inhibit cAMP production [1]. Increases in cAMP can activate different families of cAMP-binding proteins. In cardiomyocytes, these include cyclic nucleotide-gated ion channels (CNGC) [2], exchange proteins directly activated by cAMP (Epacs) [3], Popeye domain-containing (POPDC) proteins [4], and protein kinase A (PKA) [5]. The most extensively studied effector molecule of cAMP is PKA. cAMP binding to the regulatory subunit of PKA results in activation of its catalytic subunit. In cardiomyocytes, PKA-mediated phosphorylation modulates ion channels, transmembrane receptors, and regulatory proteins, leading to increased heart rate, increased strength of contraction, and enhanced relaxation. All these effects can improve haemodynamic performance in patients with a failing heart. Consequently, the cAMP signalling pathway is a unique point of interest in the development of treatments for heart failure and congenital heart disease. 

Interestingly, not all extracellular stimuli that activate cAMP signalling in the heart increase PKA activity uniformly and to a similar degree throughout the cell. This was first hypothesised on the basis of observations in biochemical fractions of cardiomyocytes, stimulated with either prostaglandins or β-adrenergic receptor agonists [6]. Direct evidence of cAMP compartmentalisation, however, had been difficult to obtain. Clean isolation of cardiomyocyte organelles, to map the heterogenic distribution of cAMP across the cell on a micrometre scale, presents a considerable challenge. Another experimental difficulty is the small molecular size of cAMP itself: cAMP occupies not more than 1.12 nm in a crystal structure in complex with PDE4D (PDB 2PW3) [7], which is more than six times smaller than the G_s_α subunit of a heterotrimeric G protein (PDB 1AZT) [8]. A small molecular radius allows for high diffusivity. In a medium with low molecular complexity, such as water, cAMP can reach a diffusion velocity of up to 444 μm^2^/s. In molecularly crowded environments, such as the cytoplasm of an adult cardiomyocyte, this can be slowed by more than a factor of ten [9]. Still, assessment of real-time signals in response to cellular stimulation is a considerable challenge when using conventional biochemical methods. To fully understand the conserved and divergent aspects of cAMP signalling downstream of distinct cellular stimuli, temporal and spatial dissection of signalling events is crucial. Live, single cell imaging methods based on fluorescence resonance energy transfer (FRET) provided the means to dissect how cellular signalling occurs in space and time. 

Real-time cAMP imaging techniques have greatly enhanced our understanding of the distribution and nature of intracellular cAMP signalling domains. Temporal and spatial resolution of cAMP signalling are among the main advantages that FRET-based imaging methods can offer to the field. In combination with the reversibility of the response and real-time detection of cAMP changes in living cells, this technique has revolutionised our understanding of cAMP signalling in the heart. FRET-based imaging helped replace the coarse definition of cAMP concentration gradients between biochemical fractions with our appreciation of sub-microscopic differences in cAMP signalling at different organelles. 

This review gives a brief account of the evolution of FRET-based imaging approaches in the study of cAMP signalling. Different probes have been instrumental in addressing specific aspects of the cAMP signalling pathway and have distinct strengths and weaknesses that are worth considering when designing experiments. We also discuss how several cardiac compartments have been characterised using real-time imaging of cAMP and present specific examples.

## 2. The Evolution of cAMP Imaging Techniques and Their Contribution to Our Understanding of the Spatio-Temporal Compartmentation of cAMP Signalling 

cAMP signalling in cardiac myocytes is compartmentalised. During a signalling event, the cyclic nucleotide is not uniformly distributed through the entire cytoplasm, but it accumulates to a greater or lesser extent in distinct loci within the cell. Due to its physicochemical properties and relatively high diffusivity, this behaviour of cAMP is not intuitive, and the discovery and further analysis of cAMP subcellular compartmentation was a multi-step process that is recounted in the following paragraphs. A single cAMP compartment can be confined to a cellular substructure that involves only a small number of proteins and thus operates on the nanometre scale. Such macromolecular substructures have been termed cAMP nanodomains. cAMP domains contain functionally associated proteins, or signalosomes, which together shape the effect of the cAMP signal within the domain. Signalosomes are usually comprised of cAMP binding proteins, such as PKA and phosphodiesterases (PDEs), as well as their regulators and targets. 

**Figure 1:**
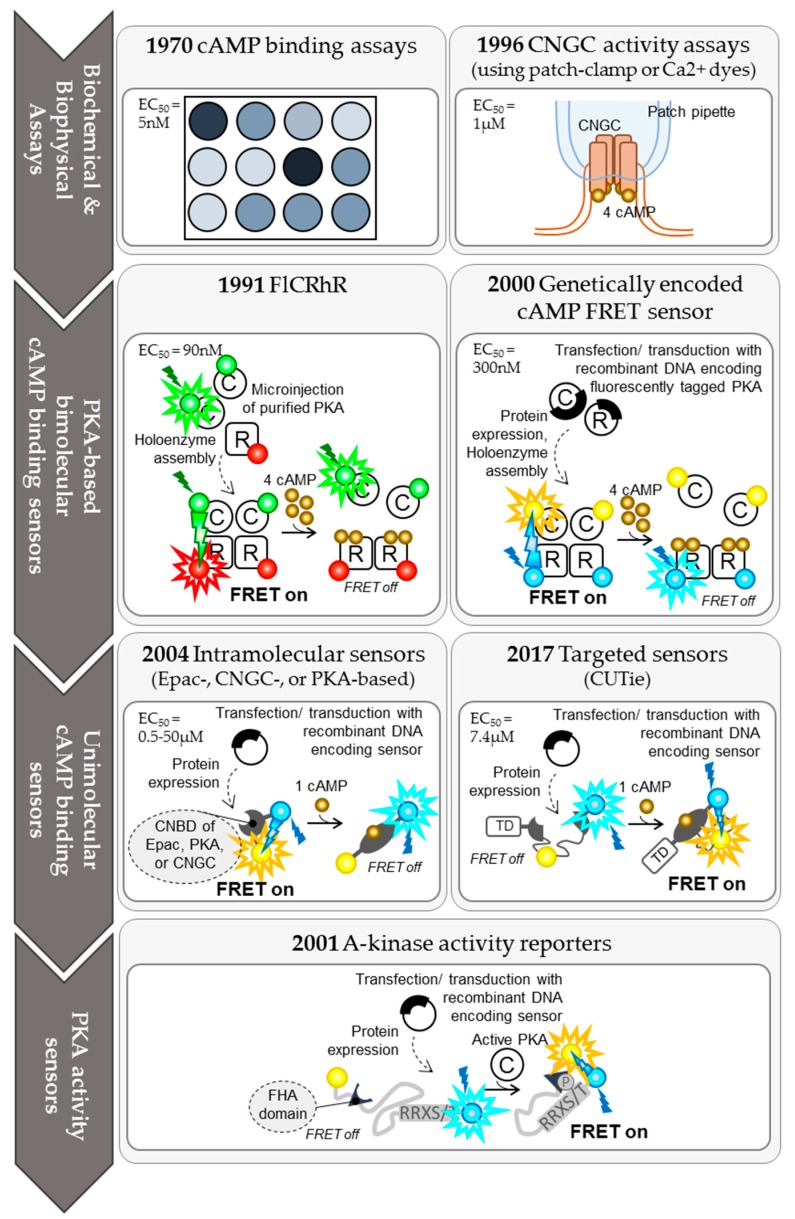
Evolution of cardiac cAMP detection from bulk biochemical analysis to targeted real-time imaging in living cardiomyocytes. In all panels, cAMP molecules are represented by golden spheres; fluorophore molecules are represented by coloured spheres, with the colours indicating their emission spectra. Exposure to light with the excitation spectrum of a fluorophore is represented by a monochrome lightning bolt; emission of fluorescent light is represented by a halo around the fluorophore. Fluorescence transfer is represented by a lightning bolt that originates from the fluorescence donor, which matches the donor in colour and points towards the fluorescence acceptor. EC_50_ for cAMP is indicated for all cAMP detection systems, as reported in the literature [10,11,12,13].

cAMP compartmentalisation has a spatial and a temporal dimension. To adequately describe cAMP signalling in cardiomyocytes, the toolkit for cAMP detection and quantification therefore had to evolve considerably from bulk analysis of cAMP content in tissue or cell lysates to targeted, real-time, and quantitative analysis of cAMP nanodomains in living cells (Figure 1). Each of the cAMP sensors described in the following has unique experimental advantages and caveats, including cAMP sensitivity, dynamic range, or sensor biology. This review aims to highlight some of the considerations to take into account when designing an experimental system.

### 2.1. Biochemical Protein Binding Assays for cAMP

Since the 1970s, detection of cAMP by calibrated amounts of cAMP binding proteins, including cAMP-dependent protein kinase purified from bovine muscle [14] or cAMP-binding antibodies [15,16], has allowed for sensitive and specific quantification of cAMP in tissue lysates, cell lysates, or fractions thereof.

Bulk biochemical quantification of cAMP in membrane and cytosolic fractions from adult rabbit cardiomyocytes revealed that selective stimulation of different families of G-protein coupled receptors elicits distinct cAMP and PKA activation profiles. Concentration of cAMP increases in both the membrane and the cytosolic fraction when β-adrenergic receptors are stimulated with isoproterenol. Meanwhile, stimulation of the prostaglandin receptor with prostaglandin E1 (PGE1) only increases cAMP in the cytosolic fraction [6].

Biochemical cAMP detection in combination with cell fractionation provided first evidence that cAMP signalling in cardiomyocytes is not uniform and that cAMP can be compartmentalised depending on the extracellular stimulus. Biochemical detection can be as sensitive as 1 nM cAMP and is compatible with high throughput screening [17]. However, the technique requires disruption of large numbers of cells. Consequently, it is limited to bulk analysis of a potentially heterogeneous population of cells, and detection of real time dynamics of cAMP is impossible.

### 2.2. Cyclic Nucleotide Gated Channel (CNGC) Activity Measurements

cAMP can activate non-selective cation channels in the plasma membrane, known as cyclic nucleotide gated channels (CNGC). Activation of these channels leads to a cation current, which can be measured using the patch clamp technique [18]. CNGC activation also triggers an increase in intracellular calcium, which can be quantified with calcium-sensitive dyes [19]. Such measurements of CNGC activity are possible at a single cell level and in living cells; thus, they overcome major limitations of biochemical techniques.

This approach added more detail to the biochemical finding that β-adrenergic and prostaglandin receptors have different effects on membrane and cytosolic cAMP levels, by revealing a temporal dimension: cAMP levels near the surface membrane transiently respond to PGE1 stimulation, while bulk cellular cAMP rises to a steady level in the same time frame [20]. Furthermore, this technique neatly couples cAMP concentration with cardiac physiology. Using patch clamp to measure CNGC-mediated cation currents revealed that β-adrenergic receptors are functionally coupled to nearby Ca^2+^ channels via local elevations of cAMP [21]. 

While CNGC-based functional assays enabled real-time detection of changes in cAMP concentrations for the first time, CNGC biosensors are restricted to one compartment, the sarcolemma. This precludes studies of other important subcellular structures in cardiomyocytes, such as the sarcomere and sarcoplasmic reticulum. In addition, native CNGCs have comparatively low cAMP/cGMP selectivity [2]. While mutagenesis improves the affinity of the channel to cAMP versus cGMP, the popular E583M single point mutant still retains some affinity for cGMP [22].

### 2.3. PKA-Based bi-Molecular FRET Sensors

#### 2.3.1. FlCRhR Probes

To create a highly selective probe that can measure cAMP in living cells, Roger Tsien, Susan Taylor, and colleagues made use of the heterotetrameric structure of the cAMP effector PKA [23]. Catalytic and regulatory subunits of PKA were expressed in bacteria as recombinant proteins, and each was tagged with chemical fluorophores of different spectral properties (fluorescein on the catalytic and rhodamine on the regulatory subunits). Upon microinjection into living cells, these labelled subunits form heterotetramers, comprising two catalytic and two regulatory subunits. Their close proximity in the heterotetramer enables fluorescence energy transfer of the fluorescein donor on one of the catalytic subunits to the rhodamine acceptor on one of the regulatory subunits. Thus, if fluorescein is excited with the appropriate excitation wavelength, energy transfer occurs within the heterotetramer, and rhodamine fluorescence emission is detectable. Upon cAMP binding, the heterotetramer dissociates, which leads to release and activation of the catalytic subunit, decrease of fluorescence energy transfer, and a concomitant decrease of rhodamine emission relative to fluorescein emission [23].

Using this probe, intracellular cAMP concentrations were measured in response to short β-adrenergic stimulation of isolated frog ventricular myocytes and correlated to the ensuing calcium transients [24]. This demonstrated that the dynamics of intracellular cAMP concentration and intracellular calcium transients are distinct from each other in response to β-adrenergic stimulation. Thus, the FlCRhR probe constituted an important step towards the dissection of temporal sequences in the cardiac cAMP signalling cascade.

While this technique allowed for specific measurement of cAMP in living cells on a single-cell level, the requirement for production of chemically labelled PKA subunits and microinjection of correctly folded proteins renders it practically challenging. Critically, both the process of microinjection and the non-physiologically high concentrations of PKA catalytic subunits in the cell produce a considerable amount of cytotoxicity that limits the number of cell types that are amenable to this technique.

#### 2.3.2. Genetically Encoded, Tetrameric cAMP FRET Probes

While FlCRhR was an important proof of concept for the use of tetrameric PKA-based FRET in living cells, there was a need to incorporate the probe into a more ubiquitously applicable genetic system. This was generated by tagging the genes for catalytic and regulatory subunits of PKA with the coding sequence for two fluorescent proteins with overlapping emission and excitation spectra. Genetically encoded FRET sensor pairs can be co-transfected into living cells, which leads to their transient expression by the host cell expression system [25]. Similar to the FlCRhR probes, an increase of cAMP leads to dissociation of the PKA heterotetramer and a decrease in energy transfer. 

This technique was widely applied to measure changes in intracellular cAMP concentrations in diverse cellular systems. Once expressed, genetically encoded PKA tetramers are compartmentalised by binding to PKA scaffolds called A-Kinase anchoring proteins (AKAPs) [26]. AKAPs can be found in many cardiac subcellular domains proximal to and distal from the sarcolemma, including the T tubules, the sarcoplasmic reticulum, and the Z lines. cAMP measurement with the PKA-based FRET probes revealed that adrenergic stimulation of neonatal rat cardiac myocytes leads to a non-homogeneous increase in cAMP concentrations, providing, for the first time, direct evidence that the activation of β-adrenergic receptors leads to the generation of multiple distinct subcellular cAMP pools. The cAMP domains aligned with AKAP-centred domains, and free diffusion of the second messenger was limited by the activity of phosphodiesterases (PDEs) [26]. The important contribution of different families of PDEs to nanodomain regulation in cardiomyocytes has recently been reviewed elsewhere [27]. Subsequent analysis of the nature of these PDEs revealed that in rat cardiac myocytes, PDE4 is the major cAMP degrading enzyme to shape amplitude and duration of cAMP responses to adrenergic stimulation. Crucially, PDE4 was shown to localise to distinct cardiomyocyte compartments that were different from other PDE isoforms, for example PDE3 [28]. A later study found that the adrenergic cAMP response over Z lines in cardiomyocytes from PDE4D^−/−^ knock out mice, as measured with the tetrameric FRET probe, was higher than in wild-type cardiomyocytes. Critically, PDE4D^−/−^ knock out mice exhibited accelerated progression of heart failure following myocardial infarction and were highly susceptible to cardiac arrhythmias during exercise followed by low-dose epinephrine injection [29].

The ability to measure real-time intracellular cAMP changes in intact, living cells also enabled studies towards understanding the integration of sympathetic and parasympathetic stimulation in cardiomyocyte cAMP signalling [30]. Using this sensor, it was demonstrated directly for the first time that termination of parasympathetic, muscarinic stimulation causes a transient increase in cAMP activity, providing important integrative data on the autonomic control of cardiac function.

Tagged with a peptide sequence that is post-translationally myristoylated and palmitoylated, the PKA-based sensor can be selectively directed to the plasma membrane [31]. In HEK cells, this strategy corroborated the biochemical finding that cAMP levels at the plasma membrane can transiently respond to PGE1 stimulation. 

The development of genetically encoded cAMP sensors has strongly facilitated the spatio-temporal dissection of cAMP signalling in cardiomyocytes. However, the multimeric nature of PKA presents several technical limitations for high temporal resolution of intracellular cAMP quantification. As catalytic and regulatory PKA subunits with acceptor and donor fluorophores are transfected and expressed from two different plasmids, equal level of expression cannot be guaranteed. Additionally, each sensor subunit can potentially interact with endogenous PKA subunits, limiting the number of functioning FRET pairs. Both these effects may skew the formation of FRET-competent heterotetramers and make signal termination difficult to assess. As dissociation of the PKA heterotetramer requires cooperative binding of four molecules of cAMP to the two regulatory subunits, sensor kinetics are relatively slow, which may lead to underestimation of cAMP propagation within the cell [32]. PKA tetramers are compartmentalised by binding to AKAPs [26] and/or due to their anisotropic diffusion [24], which limits their applicability to total cytosolic cAMP measurements in cardiomyocytes. Lastly, while the endogenous expression system limits the amount of PKA produced, the tagged catalytic subunits of the sensor retain catalytic activity. This slightly elevated PKA activity can still be toxic in some cell types [33]. However, this remains the only available sensor that can directly report on kinetics of PKA activation in intact cells [34].

### 2.4. Intramolecular FRET Sensors

To overcome cooperativity and remaining toxicity concerns, single-chain cAMP FRET sensors were developed using the cyclic nucleotide binding domains (CNBDs) obtained from a number of different cAMP binding proteins.

#### 2.4.1. Epac-Based Single-Chain FRET Sensors

Several generations of unimolecular FRET sensors based on exchange protein directly activated by cAMP (Epac) are available to date. Epac is a guanine nucleotide exchange factor (GEF) for Ras-like small GTPases. Binding of cAMP to a unique binding domain induces a conformational change in the inactive Epac protein to expose both the catalytic domain [35] and a targeting domain [36], stimulating GEF activity. In Epac-based sensors, fluorescence donor and acceptor proteins are fused to the N- and C-terminus of either a single cAMP binding domain of human Epac1, a single cAMP binding domain of murine Epac2, full length human Epac1, or truncations of human Epac1 [32,37,38]. While the initial sensors had a limited dynamic range, giving between 10% and 30% FRET change, their range has been significantly improved to over 150% by sequential engineering of both the spectral properties of the FRET pairs and the way they are assembled with the CNBDs [39,40,41].

The uniform cytosolic distribution, particularly of the sensors based on a single cAMP binding domain, uncoupled the site of cAMP detection from any particular cellular structure and allowed for detection of bulk cytosolic cAMP. However, the site of cAMP detection could be chosen deliberately by fusing these sensors to specific targeting sequences or domains. Directing the sensor to nuclei, mitochondria, or the mitochondrial matrix, for example, allowed for measurement of the dynamic local changes in cAMP concentrations in response to PGE1 or adrenergic stimulation [37], revealing differential dynamics of cAMP signalling in response to the activation of either receptor. Using these sensors coupled to the targeting domain from different PKA isoforms demonstrated that in cardiac myocytes, compartmentalised PKA-RI and PKA-RII respond to distinct, spatially restricted cAMP signals, which leads to phosphorylation of unique subsets of downstream targets [42].

An Epac-based single-chain FRET sensor, in its untargeted form, was used in combination with scanning ion conductance microscopy (SICM). After local receptor stimulation of either β1- or β2-adrenergic receptors, cAMP was found to be differently distributed across the healthy cardiomyocyte membrane. As a consequence of β2-adrenergic receptor redistribution in heart failure, such cAMP compartmentation was altered [43].

Epac-based sensors are versatile and easy to use, but their properties may need to be adjusted to suit a particular cellular system. Some of the Epac-based sensors, including the popular Epac1-camps, have high cAMP sensitivity (ca. 1 μmol/L, [32]). This can lead to fast saturation and limited coverage of the physiological cAMP concentration spectrum, especially in cells with high basal cAMP levels such as cardiomyocytes. Furthermore, the spectral properties of the ECFP/EYFP-containing sensors are relatively sensitive to changes in the intracellular microenvironment, for example intracellular pH [44], which has been improved in later versions of the sensors [41]. However, the critically limiting factor of this system pertains to its molecular design. In all the targeted versions of this sensor [37,42,45,46,47], the targeting domain is directly attached to one of the fluorophores. This affects the FRET properties that depend on the targeting domain of choice, making direct comparison of cAMP signals detected at different intracellular sites difficult [12].

#### 2.4.2. CNGC-Based Single-Chain FRET Sensors

The sensitivity of Epac-camps sensors to cAMP is a concern in cardiomyocytes with high basal cAMP concentrations, and so a sensor that sandwiches an alternative cyclic nucleotide binding domain between its fluorophores was developed. Here, fluorescence donor and acceptor proteins are fused to the cAMP binding domain of the hyperpolarization-activated, cyclic, nucleotide-gated potassium channel 2 (HCN2) [33].

With this sensor, contributions of different PDE isoforms to cAMP hydrolysis in adult mouse cardiomyocytes after adrenergic stimulation were assessed, which ranked the activity of PDE4 above PDE2 and PDE3 [33]. Measuring cAMP propagation after selective stimulation of β1- and β2-adrenergic receptors indicated that cAMP signals that emanated from β1 receptors propagate over a distance involving multiple sarcomeres in adult cardiomyocytes. In contrast, the cAMP signal on β2 stimulation remained strictly confined [33].

While this sensor addresses some limitations of the Epac-based cAMP sensors in cardiomyocytes, the main issue of comparability between targeted sensors remained unresolved.

#### 2.4.3. PKA-Based Single-Chain FRET Sensors

To minimise potential interference of the targeting domain with FRET, a novel cAMP sensor named CUTie (cAMP Universal Tag for imaging experiments) was developed based on the CNBD of the PKA regulatory subunit IIβ. The unique feature of this new sensor is that CUTie enables fusion of the targeting domain distal to the FRET fluorophore pair. This is attained by fusion of the FRET donor to the C-terminus of the CNBD and insertion of the FRET acceptor in an intra-domain loop of the CNBD, leaving the N-terminus free for the targeting domain. In this configuration, targeting domain and FRET module are physically separated from each other and steric hindrance on the conformational change required for energy transfer is minimised. As a result, the dynamic range of the sensor in different compartments is now comparable [12]. This is not necessarily the case for other reporters that have been targeted to subcellular sites. For studies involving targeted sensors, it is important to keep in mind that their cAMP-binding and spectral properties can be influenced by the targeting domain and its impact on the overall fold of the polypeptide chain. Comparison of the cAMP response at different sites must therefore be interpreted with caution, unless accurate calibration curves are available for the targeted reporters.

The demonstrable independence of the sensor FRET response from the chosen targeting domain, in combination with in-cell calibration techniques, paved the way for accurate quantitation of cAMP concentrations at different compartments of healthy and hypertrophic myocytes. Direct quantitative comparison of cAMP levels obtained with CUTie sensors, targeted to plasmalemma, sarcoplasmic reticulum, or sarcomere, suggested that the size of cAMP subcellular compartments can be sub-microscopic. Importantly, it was observed that the amplitude of the local cAMP signal is independent of the distance from the site of cAMP synthesis. It was also dependent on the activity of phosphodiesterases, indicating that compartmentalisation of cAMP is not simply the result of limited diffusion due to the complex intracellular structure but is an actively regulated phenomenon. The study also showed that the effect of phosphodiesterases is most profound at the sarcomeric nanocompartment. Application of targeted CUTie reporters that were used to compare healthy and diseased hearts revealed that nanodomains can be differentially affected by misregulation of β-adrenergic signalling in heart failure [12]. This highlights the opportunities inherent to selective targeting of cardiac compartments in heart failure therapy. Focussing therapeutic interventions on the compartments that are selectively affected in heart failure may reduce the adverse outcomes of current inotropic agents in long-term treatment [48].

### 2.5. Single-Wavelength Fluorescent Sensors for cAMP

In cardiomyocytes, cAMP signalling is closely linked to calcium signalling. It is possible to use ECFP/EYFP FRET sensors in combination with Fura-2, a calcium imaging dye with absorption peaks at 340 nm (Ca^2+^-bound) and 380 nm (Ca^2+^-free), and follow the activation of both pathways simultaneously [49]. To streamline such multi-colour measurements for off-the-rack microscope setups, several single-wavelength sensors for cAMP have been developed. In Flamindo and Flamindo2, the cAMP binding domain of Epac1 is sandwiched between two halves of the EYFP variant Citrine. Binding of cAMP decreases Citrine fluorescence intensity [50]. In the newly developed cAMPr sensor, circularly permuted GFP is flanked by the full-length catalytic subunit of PKA on one, and a regulatory subunit of PKA lacking the dimerization/docking domain on the other, side. Binding of cAMP separates the PKA subunits and increases GFP fluorescence [51]. Both sensors have been used in combination with calcium sensing dyes, albeit not in cardiomyocytes.

### 2.6. PKA Activity Sensors

The most extensively studied effector of cAMP in the heart is protein kinase A (PKA) [52]. A family of single-chain FRET sensors was developed to assay PKA-mediated phosphorylation in cells. In these A-kinase activity reporters (AKARs), a phosphorylatable PKA consensus sequence is combined with a phosphate-binding domain into a single polypeptide chain. The two domains of the sensor are flanked by a FRET pair of fluorophores [53]. Phosphorylation of the PKA consensus leads to the interaction of the phosphate-binding domain with the now phosphorylated sequence, which mediates the conformational change necessary for FRET to occur. Since its first conception, the sensor has been optimised in several rounds to give a maximum FRET efficiency of 60% in the latest version [54,55,56]. An important feature of later versions of this sensor is that it allows for dephosphorylation of the probe. As such, these sensors provide information on both PKA activity, which increases FRET, and phosphatase activity directed against PKA targets, which decreases FRET [54]. 

Similar to cAMP probes, these sensors are theoretically targetable to any cellular compartment or signalosome. As such, they are useful reporters of the functional effects of cAMP signalling on effector proteins within a pre-defined compartment. Tethering of AKAR reporters to AKAP-binding domains, for example, shortened the response time of the sensor to adenylate cyclase stimulation, whereas tethering to a nuclear localisation sequence prolonged it [53]. This emphasised the importance of proximity in determining which substrates are preferentially phosphorylated by the anchored PKA kinase.

The use of AKAR reporters is extremely effective in real-time quantification of PKA-mediated phosphorylation in a given cellular compartment. It is important to keep in mind that this is an indirect measure of the cAMP signal in this compartment, as signal intensity not only depends on the cAMP concentration but also on the availability of functional PKA to mediate AKAR phosphorylation, and on the level of counteracting phosphatase activity.

## 3. Cardiac Nanodomains Studied Using FRET-Based cAMP Sensors

**Figure 2:**
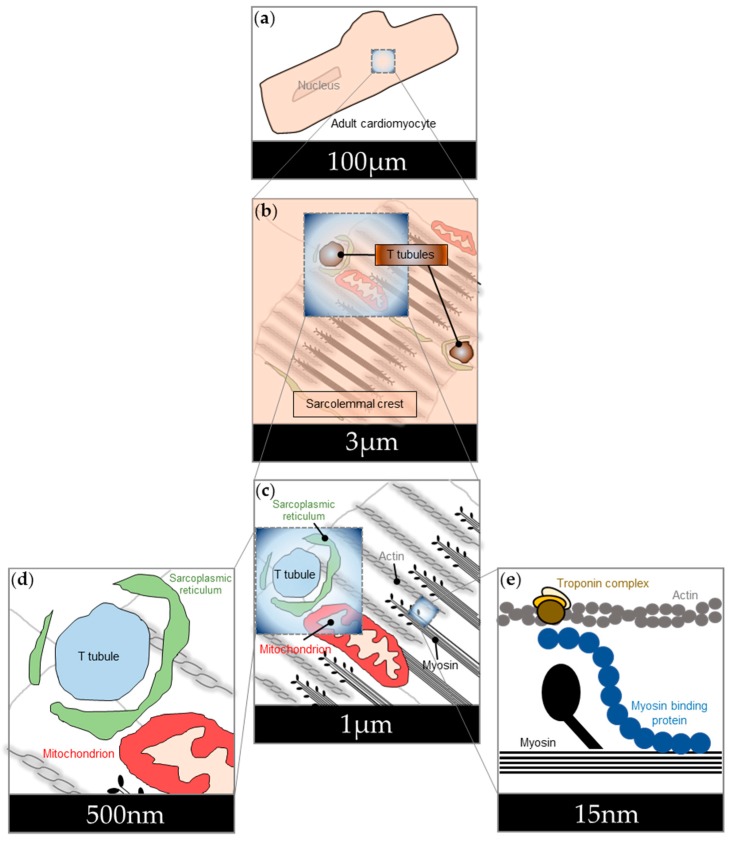
Scales of cAMP signalling domains in adult cardiomyocytes. Continued development of cAMP detection methods improved the resolution of cAMP signal measurement within adult cardiomyocytes. (**a**) Initially, whole-cell and fractionation assays across a 100 μm long adult cardiomyocyte were performed. (**b**) Later, differences in cAMP concentrations were measured along the sarcolemmal crest and T tubules, which are spread across the membrane roughly every 2 μm. (**c**) Targeted sensors showed differential cAMP signalling between cytosol and mitochondrial matrix, which can be located under 1 μm apart from each other in cardiomyocytes. (**d**) Even differences between mitochondrial outer membrane and matrix, or sarcoplasmic reticulum and T tubules, in cardiomyocytes ca. 100 nm apart, can be detected. (**e**) Future sensors might be able to quantify signalling events within single signalosomes that are few nanometres apart, such as the protein complexes of the sarcomere.

Development of genetically encoded cAMP sensors greatly enhanced our ability to describe the biochemical properties of cAMP in living cells through real-time visualisation of cAMP responses. The sensors allowed for more accurate definition of the diffusive properties of the small signalling molecule in a cellular context. Physical barriers in the structurally highly complex adult cardiomyocytes, for example, considerably slow down diffusivity of cAMP compared to the loosely structured neonatal cardiomyocyte [9]. Accurate physiological calibration of the sensors also enabled precise measurement of cAMP binding to cAMP-dependent proteins, such as PKA, in their actual intracellular context [57]. Importantly, genetically encoded cAMP sensors were used to demonstrate, for the first time, that gradients of cAMP are formed within intact cardiac myocytes upon adrenergic stimulation [26]. The possibility to target FRET sensors to distinct subcellular structures makes them an ideal molecular tool for monitoring confined intracellular cAMP nanodomains (Figure 2). cAMP nanodomains often contain a combination of PKA targets and regulators, as well as domain-specific PKA anchoring proteins, PKA itself, and phosphodiesterases. We now know that each nanodomain comprises a unique set of, and combination of, these proteins. PKA targets can be structurally and functionally diverse, including, for example, mechanoenzymes [58], structural proteins [59], regulatory proteins [60], or ion channels [61]. There are at least forty known PKA anchoring proteins, and new ones are still being discovered [62,63]. Depending on the type of regulatory subunit, PKA complexes themselves are targeted to different subcellular sites [64]. Phosphodiesterases are a diverse family of enzymes that can be grouped into 11 families (PDE1-PDE11), with each family often containing distinct variants that are differentially expressed and localised [65]. FRET-based cAMP sensors have been instrumental in detailing the components of signalosomes in different compartments and thus in defining biochemical and physiological effects of local cAMP signalling in cardiomyocytes.

### 3.1. cAMP Signalling in the Nucleus

Transcriptional regulation of nuclear genes can alter metabolic and developmental programmes, and changes in transcriptional profile are strongly associated with pathological conditions, such as cardiac hypertrophy [66]. Major targets of the cAMP signalling pathway in the nucleus are transcription factors, including the Nuclear factor of activated T-cells (NFAT) and cAMP response element-binding protein (CREB) families, as well as Class II histone deacetylases (HDACs), which regulate gene transcription by increasing the compaction state of DNA. Transcription factors and HDACs can shuttle between nucleus and cytoplasm. Whether cAMP signalling affects them inside the nucleus, outside, or both, is not fully resolved. This is particularly interesting, because G-protein coupled receptors couple predominantly to plasma membrane adenylate cyclases (pmACs), i.e., the primary site of cAMP production after their activation is the plasma membrane. Hence, for nuclear targets of the cAMP pathway, this production site of the second messenger is considerably far removed from their site of activity (Figure 2a). However, there is a soluble adenylate cyclase (sAC) that localises to the nucleus [67,68], raising interesting questions about origin and propagation of cAMP in relation to nuclear signalling.

As detected by Epac-based cAMP FRET sensors targeted with a nuclear localisation sequence (NLS), cAMP concentration in the nucleus increases after stimulation of β-adrenergic or prostaglandin receptors in human embryonic kidney (HEK 293) and airway smooth muscle (ASM) cells [31,37,69]. The increase in cAMP in this compartment reached a plateau after 2 min. However, the increase in cAMP was not immediately followed by an increase in PKA substrate phosphorylation, as measured with the functional PKA sensor AKAR, likewise targeted to the nucleus. Instead, the AKAR response showed a delayed onset of 5–10 min, giving a plateau only after 30 min [37,55]. This delay was inconsistent with activation of resident nuclear PKA by cAMP after β-adrenergic stimulation. It instead favoured a hypothesis that cytoplasmic PKA catalytic subunits, activated in the cytoplasmic compartment, need to translocate to the nucleus to phosphorylate any nuclear targets. In a different study, exclusive nuclear targeting of sAC along with nuclear AKAR revealed that cAMP generated directly in the nucleus of HEK 293 cells can, in fact, activate nuclear PKA immediately after stimulation [70]. Interestingly, even the transmembrane adenylate cyclases can activate nuclear PKA activity much faster, provided PDEs, particularly PDE4, are inhibited, or PKA anchoring to AKAPs is prevented [70]. 

The direct mechanism of nuclear PKA activation does not seem to be conserved in cardiomyocytes. PDE inhibition in neonatal rat ventricular myocytes does not accelerate nuclear AKAR phosphorylation, despite rapid nuclear cAMP accumulation after co-treatment with adenylate cyclase activators and PDE inhibitors [71]. Disruption of PKA anchoring likewise does not enhance the kinetics of nuclear PKA activity. This suggests that, in cardiomyocytes, translocation from the cytoplasm is the limiting step in nuclear PKA activity. This mechanism may help prevent onset of transcription factor-mediated hypertrophy during transient sympathetic activation.

### 3.2. cAMP Signalling at the Sarcolemma

The sarcolemma has distinct microscopic domains. T tubules (Figure 2b) reach deep into the cardiomyocyte, increasing its surface area and facilitating rapid delivery of extracellular ions to the core of the cardiomyocyte. Moreover, T tubules contain unique proteins compared to other membrane areas, and they form close contacts with the sarcoplasmic reticulum (Figure 2c,d). Both features cause unique signalling events to take place in T tubular membrane sections. Another smaller membrane compartment, which is best visualised in electron microscopy images, is specialised membrane pits called caveolae. Caveolae, too, increase the membrane surface area and facilitate the formation of signalling complexes. 

Using plasma membrane-targeted cAMP sensors, it was established that proximity to the plasma membrane can have a temporal effect on the onset of cAMP signalling. For example, in one study the response time of the Epac-based cAMP sensor ICUE1 targeted to the plasma membrane was 40% reduced over its diffuse cytoplasmic counterpart upon β-adrenergic stimulation [37]. To address the functional effects of such differences in signal initiation, plasma membrane targeted and untargeted AKAR probes were compared in neonatal cardiomyocytes [72]. This study showed that PKA phosphorylation gradients in response to local cAMP production depended predominantly on restricted cAMP diffusion, PDE-mediated cAMP degradation, and PKA-mediated cAMP buffering. Moreover, the different effects of prostaglandins and adrenergic stimulation on cAMP signalling in cardiomyocytes were further described in this study. Prostaglandins stimulated higher PKA activity in the cytosol than at the sarcolemma, whereas β-adrenergic stimulation triggered faster sarcolemmal responses than cytosolic [72].

Not only the agonist, but also the receptor subtype affected the propagation of cAMP in cardiomyocytes. While β1-adrenergic stimulation lead to Epac-based cAMP detection throughout the entire cell, β2-adrenergic stimulation responses were locally confined by an unknown mechanism [33]. β2-adrenergic signalling was similarly confined in human embryonic kidney (HEK 293) cells [40]. Here, β2-adrenergic signalling detected by an untargeted Epac-based sensor, ICUE3, could be amplified by disrupting membrane rafts through cholesterol depletion. In cardiomyocytes, β1-adrenergic receptors can be found in both caveolar and extra-caveolar fractions of the sarcolemma, while prostaglandin receptors are excluded from caveolar fractions. Disruption of caveolae by cholesterol depletion did not lead to significant differences in cytosolic cAMP responses for either of these receptors [73], indicating that the extracaveolar receptor fraction dominates cAMP production in cardiomyocytes. A study combining Epac-based cytosolic cAMP sensors with SICM confirmed that β1-adrenergic receptors can be found across the entire sarcolemma. Yet, β2-adrenergic receptors were exclusively localised to T tubules [43]. Critically, this functional compartmentation is perturbed in failing hearts, leading to loss of the restricted nature of the β2-adrenergic cAMP signal. Later, it was shown using the same technique that caveolin 3 promotes compartmentation of β2-adrenergic cAMP signalling to the T tubules, and overexpression of caveolin 3 in failing cardiomyocytes can partially restore the delocalised signal [74].

Downstream of β1-adrenergic signalling, but not β2-adrenergic signalling, PDE4B-mediated degradation of cAMP is activated in cardiomyocytes [75]. PKA inhibition in wild type, but not PDE4B knock out myocytes, triggered a significant increase of adrenergic receptor generated cAMP levels in the sarcolemmal compartment, indicating that a combination of PKA and PDE4B activity is required to maintain physiological cAMP levels in the β1-receptor domain. These FRET measurements with a plasma membrane-targeted Epac-based sensor thus provided first evidence for a localised, β1-adrenergic receptor-coupled feedback mechanism in cardiomyocytes. This feedback proved essential in the regulation of the amplitude of intracellular calcium currents after adrenergic stimulation. Calcium levels after adrenergic stimulation were higher in PDE4B knock out mice than in controls, which highlights the delicate balance in cAMP production and degradation that healthy cardiomyocytes must maintain in response to sympathetic activation.

### 3.3. cAMP Signalling at the Mitochondria

In adult cardiomyocytes, mitochondrial fatty acid beta-oxidation is the major source of energy. For this reason, cardiomyocytes are tightly packed with mitochondria (in a healthy heart, about 95% of the heart’s ATP production takes place there [76]). This energy is needed to fuel sarcomeric contraction and ATP-dependent ion flux between compartments. Heart failure is associated with significant reductions in mitochondrial respiratory capacity and mitochondrial membrane disruption [77].

cAMP signalling plays important roles in mitochondrial calcium handling, mitochondrial metabolism, and protection against cytochrome c-mediated apoptosis in cardiomyocytes. Close proximity (Figure 2c,d) between cardiomyocyte mitochondria and sarcoplasmic reticulum facilitates calcium-dependent regulation of mitochondrial respiration upon sympathetic activation. Metabolic activity of the muscle cell is thus tightly synchronised with myocyte activation [78,79,80,81]. 

Ischaemia-reperfusion injury of the heart is associated with a dramatic change in mitochondrial morphology. Changes in mitochondrial structure impact their function and have been shown to induce cyotochrome c-dependent cardiomyocyte apoptosis [82]. Interestingly, cAMP signalling after pharmacological β-adrenergic stimulation or stress and exercise could counteract this programme. [83].

To specifically describe mitochondrial cAMP signalling, FRET-sensors were fused to different mitochondrial targeting domains. An Epac-based FRET sensor, fused to the mitochondrial PKA scaffold protein AKAP1, showed a similar FRET response to β-adrenergic stimulation as in the cytosol [37]. AKAP1 binds to the outer mitochondrial membrane and faces the cytosol [84]. The same sensor was targeted to the mitochondrial matrix, using the first 12 amino acids of human cytochrome oxidase c (subunit IV). The matrix-targeted sensor responded to β-adrenergic stimulation, initially suggesting that membrane-generated cAMP can enter mitochondria and activate downstream signalling in the matrix [37]. This view of a continuous cAMP gradient shared between cytosol and mitochondria was contested by observations made with AKAP-targeted PKA activity sensors. Baseline phosphorylation of the sensor was much higher than its cytosolic equivalent, and phosphodiesterase inhibition even enhanced this difference [55]. Therefore, the cytosolic surface of mitochondria seemed to regulate downstream cAMP signalling differently from the cytosol. This was confirmed with a later generation of PKA activity sensors tagged with the OMM-targeting peptide yTOM70 [85]. When PKA-mediated phosphorylation after extracellular signal termination was monitored in this system, phosphorylation at the mitochondria persisted for longer than in the cytosol. The corresponding outer membrane cAMP concentrations, studied with an OMM-targeted Epac-based sensor, on the contrary, mirrored the bulk cytosol. A matrix-targeted sensor, however, was unable to detect cAMP generated at the plasma membrane. Two separate studies showed that cAMP is unable to permeate the inner mitochondrial membrane [45,85], although both groups found evidence for cAMP signalling within the mitochondrial matrix. The discrepancy between the initial study and follow-up research is most likely due to imprecise targeting of the first sensor. While the authors could demonstrate that some of the sensor was indeed localised to the mitochondrial matrix [37], a significant proportion remained cytosolic [37,45]. This provides key learnings for the design of targeted FRET sensors. It highlights the importance of exclusive targeting of the sensor to the compartment of interest.

In contrast to cAMP generated at the sarcolemma, calcium release from the ER or capacitative calcium influx from the extracellular medium could activate the matrix cAMP sensor [45]. The soluble adenylate cyclase activator bicarbonate, similarly, increased both matrix cAMP concentrations [45] and matrix PKA phosphorylation [85]. As a result, cAMP in the matrix is now thought to be generated by a mitochondrial sAC. The cAMP FRET response in the matrix was further increased by panspecific inhibition of phosphodiesterases, suggesting that matrix cAMP is modulated by phosphodiesterases. Functionally, cAMP signalling in the mitochondrial matrix domain enhances mitochondrial metabolism. Activation of sAC or treatment with phosphodiesterase inhibitors increased mitochondrial ATP production, as measured in a mitochondrially-targeted luciferase assay. PKA inhibition reduced mitochondrial ATP production [45].

Using OMM-, and matrix- and untargeted Epac-based sensors, the outer mitochondrial membrane cAMP nanodomain was subsequently dissected further. It was confirmed that this domain on the mitochondrial surface is supplied by plasma membrane adenylate cyclases and is independent of any cAMP generated in the mitochondrial matrix by sAC [86]. Instead of regulating mitochondrial metabolism, this pool of cAMP is involved in regulation of mitochondrial dynamics and protects them from ionomycin-induced apoptosis.

### 3.4. cAMP Signalling at the Sarcoplasmic Reticulum

The sarcoplasmic reticulum (SR) of mammalian adult cardiomyocytes is a highly differentiated organelle and the main regulator of calcium transients in these cells. Its proximity to cadiomyocyte T tubules (Figure 2c,d) facilitates rapid translation of adrenergic activation in the T tubular membrane compartment to induction of calcium ion (Ca^2+^) currents from the SR. Cardiac Ca^2+^ homeostasis is vital in maintaining the ability of the cardiomyocyte to contract and relax, as Ca^2+^ is a necessary co-factor for the force-generating module in the sarcomere [87]. 

Calcium cycling at the SR is heavily regulated by PKA. PKA phosphorylates the channels that release Ca^2+^ from the SR to enable contraction (ryanodine receptors, RyR) [61,88], as well as phospholamban (PLN), a protein that regulates the sarco/endoplasmic reticulum Ca^2+^-ATPase (SERCA) [89]. Ca^2+^ handling by the SR is often dysfunctional in heart diseases, including heart failure [90].

An Epac-based cAMP sensor, fused to full-length phospholamban using a flexible linker, was used to investigate propagation of adrenergic signals to the SR. The SR compartment was found to be predominantly under β1-adrenergic control [46]. β2-adrenergic signals were not strong enough to produce a cAMP increase at the SERCA microdomain that was detectable with the phospholamban-anchored sensor. β1-adrenergic stimulation, on the other hand, produced a cAMP signal at the SR that even surpassed the cytosolic cAMP signal in amplitude. The robust effect of β1-adrenergic stimulation in the SR compartment was corroborated in rabbit cardiomyocytes using a PKA activity sensor targeted to the SR with only the transmembrane domain of phospholamban [91]. Interestingly, this sensor also detected a small but significant change in PKA activity upon β2-adrenergic stimulation, although this signal was significantly lower than after β1-adrenergic stimulation [91]. This could mean that either cAMP compartmentalisation differs slightly between species or the amplification of the cAMP signal by PKA is necessary to render the signal detectable. Pan-specific adrenergic stimulation induces a rapid and significant increase in both cAMP concentrations and PKA activity at the SR, as measured by a CUTie sensor targeted to the SR with full length AKAP18δ or the phospholamban PKA activity sensor [12,91,92]. Direct pharmacological activation of adenylyl cyclases induced much slower and smaller increases in PKA-mediated phosphorylation at the SR than activation of the cyclase following adrenergic stimulation, raising the question of how the adrenergic signal is more efficiently relayed to the SR compartment [92]. 

This could be increasingly relevant as transverse aortic constriction (TAC), which causes pressure overload-induced cardiac hypertrophy in the heart, reduces any differences in cAMP between SR and cytosol observed after adrenergic stimulation [46]. Phosphodiesterases act to counterbalance loss of cAMP gradients. The adrenergic cAMP concentration gradient between SR and cytosol can be reduced upon additional inhibition of phosphodiesterases [46]. This was confirmed when cAMP concentrations at the SR were compared to nanodomains at the sarcolemma or sarcomere using CUTie sensors fused to full length AKAP18δ (SR), AKAP79 (sarcolemma), or cardiac troponin I (sarcomere) [12]. While low-level, pan-specific adrenergic stimulation resulted in different concentrations of cAMP in each compartment, this difference was completely abolished by concomitant inhibition of phosphodiesterases. Measurement of PKA activity in the same compartments with a PKA activity sensor fused to the transmembrane domain of phospholamban (SR), a membrane targeting peptide from Kras (sarcolemma), or the C-terminus of troponin T (sarcomere) confirmed differential regulation of these compartments for β2-adrenergic signals [91]. Strikingly, phosphodiesterase inhibition levelled cAMP concentrations, even in diseased hearts, in which adrenergic responses in the SR and sarcomeric compartments were dampened [12]. Differential analysis of the contribution of specific families of phosphodiesterase revealed that their contribution to reducing phosphorylation in the three compartments varies depending on the health of the myocyte [91]. Both of these results emphasise the importance of local phosphodiesterases in shaping cAMP gradients in both normal and disease conditions.

### 3.5. cAMP Signalling at the Sarcomere

The sarcomere is the proteinaceous force generator within the cardiomyocyte that mediates muscle contraction. It contains more than 600 structural, regulatory, and associated proteins [93]. The main functional units of the sarcomere are interlaced actin and myosin filaments (Figure 2c). The sarcomere contains some of the most-studied PKA targets in the cardiomyocyte (Figure 2e). PKA phosphorylation at the sarcomere increases contractility by enhancing actin-myosin cross-bridge formation by cardiac myosin binding protein [94]. PKA also mediates relaxation via troponin I phosphorylation by reducing the affinity of the troponin complex for Ca^2+^ [60,95]. In doing so, it ensures the speedy recovery of the sarcomere in preparation for the next cycle. 

Taking into account the important functional role of PKA activity in sarcomere contraction, it is interesting that this cellular compartment undergoes the smallest change in cAMP in response to adrenergic signalling in healthy cardiomyocytes. Comparing cAMP levels quantified with CUTie sensors targeted to the sarcomere with full length cardiac troponin I to sarcolemmal and SR-targeted sensors, low level adrenergic stimulation generated only about 60% as much cAMP at the sarcomere than at the other compartments [12]. Calibration of the sensor allowed translation of the measured FRET value to PKA activity, and it was estimated that the amount of cAMP measured at the sarcomere with the CUTie sensor would only activate PKA to about 5% of its maximal activation, in which the activity in resting cells was measured to be ca. 3% [12]. This correlates well with measurements in rabbit myocytes using sarcomere-targeted PKA activity sensors [91], in which the sarcomere was shown to be less sensitive to adrenergic stimulation than sarcolemma and sarcoplasmic reticulum. For example, in cells exclusively stimulated through the β2-adrenergic pathway, sarcomeric PKA phosphorylation was barely detectable by FRET, whereas sarcolemma and sarcoplasmic reticulum showed robust responses [91]. Both the mouse and the rabbit studies show that inhibition of PDEs elevates cAMP and PKA signalling at the sarcomere.

### 3.6. cAMP Signalling at A Kinase Anchoring Proteins (AKAPs)

AKAPS are intracellular scaffolding proteins that provide a platform for the assembly of PKA-containing signalosomes. AKAPs were first discovered in the 1970s and 80s [96] and then catalogued in different tissues using interaction screens with the PKA dimerization/docking domain that contained an AKAP-binding consensus [97,98]. 

Targeting FRET sensors to distinct AKAPs can be utilised to directly describe cAMP fluctuations around defined PKA signalosomes in cardiomyocytes. CUTie sensors were used to quantitatively compare cAMP concentrations in the vicinity of AKAP79 at the plasma membrane or AKAP18δ at the sarcoplasmic reticulum with cytosolic cAMP concentrations after adrenergic stimulation of cardiomyocytes. These studies revealed that cAMP concentrations around AKAPs can be detectibly higher than in the bulk cytosol [12].

AKAPs interact with PKA through AKAP-binding domains on the regulatory (R) subunits. Of note, different isoforms of R subunits preferentially interact with distinct AKAPs. By tethering Epac-based cAMP reporters to the dimerization/docking domains of RI and RII, it was possible to specifically dissect molecular components that shape cAMP pools around these different PKA isoforms in cardiomyocytes [42]. Both compartments have access to cAMP at similar levels after direct activation of adenylate cyclase. However, β-adrenergic stimulation leads to a stronger increase in cAMP at the PKA-RII containing compartments, whereas glucagon-like peptide, glucagon, and prostaglandins activate cAMP signalling around PKA-RI. These differences were abolished by simultaneous inhibition of all major phosphodiesterases, highlighting their critical role in shaping distinct receptor-mediated cAMP responses. More detailed analysis of the two compartments by combining β-adrenergic receptor stimulation with family-specific phosphodiesterase inhibitors revealed that, while PDE2 inhibition affected cAMP concentrations in both the RI and the RII compartments, PDE3 inhibition only increased cAMP in the RI compartment [99]. Using a targeted PKA activity sensor confirmed that elevated cAMP levels after co-treatment with β agonist and PDE2 inhibitor lead to increased PKA-dependent phosphorylation in the RII compartment versus the RI compartment. This is significant, because PDE2 inhibition in cardiomyocytes that are chronically stimulated with a β agonist prevented their hypertrophic growth through PKA-mediated phosphorylation of NFAT [99]. Phosphorylation inhibits translocation of the transcription factor to the nucleus and thus activation of the hypertrophic gene transcription programme under its control [100,101]. A different study with the RI and RII-targeted cAMP sensors found that crosstalk with other signalling pathways could help shape the cAMP signal in PKA compartments [102]. Local co-stimulation of cyclic GMP signalling after adrenergic stimulation, for example, selectively reduces the cAMP response around PKA-RII, levelling cAMP signal intensity in PKA-RI and PKA-RII compartments. This study highlights the value of FRET-based cAMP detection, not only for describing the cAMP pathway in isolation, but also for addressing signal integration with other simultaneously active pathways in cardiomyocytes.

Genetically encoded, tetrameric PKA-RII containing FRET probes naturally localise to AKAPs in cardiomyocytes. These probes identified a prominent role for PDE2 in shaping the cAMP response to catecholamines [103]. Inhibiting PDE2, while quantifying cAMP with the anchored FRET sensors, suggested tight coupling of PDE2 to the pool of adenylyl cyclases activated by β-adrenergic receptor stimulation. This coupling resulted in a feedback control loop, in which activation of β3-adrenergic receptors counteracted cAMP generation by β1/β2-adrenoceptors.

To dissect the role of AKAPs on cAMP signal compartmentation themselves, cytosolic FRET responses were compared in neonatal cardiac myocytes from wild type and AKAP5 knock out mice [104]. While there was a clear difference in the amount of cAMP detected after selective β1- or β2-adrenergic stimulation in the wild type, AKAP5 knockout myocytes produced equal cytosolic cAMP levels in response to β1- or β2-adrenergic stimulation.

## 4. Conclusions

The use of FRET-based cAMP and PKA activity sensors has greatly enhanced our understanding of spatial organisation in the cAMP signalling cascade, as well as the mechanisms that generate a uniquely patterned cAMP response through cardiomyocytes as they react to defined stimuli. 

Localised FRET sensors have refined our ability to monitor cAMP signalling events. The sensors constantly evolve (Figure 1), thus increasing the resolution of cAMP monitoring around distinct signalosomes (Figure 2). Initial biochemical studies drew attention to the cAMP concentration gradients between sarcolemma and bulk cytosol of adult cardiomyocytes. This was later confirmed by CNGC activity assays and FRET. cAMP measurements with FRET-based probes, especially in combination with other powerful techniques such as SICM, allowed for narrowing down single cAMP signalling domains to individual T-tubules, which are spread across the sarcolemma of adult cardiomyocytes ca. 2 μm apart from each other [43]. Development of targeted sensors first highlighted differences in cytoplasmic and mitochondrial matrix cAMP concentrations [45,85,86], i.e., in compartments that are only few micrometres apart [105]. This was further refined by sensors targeted to different compartments of the mitochondria itself [85,86]. Likewise, sensors targeted to the sarcoplasmic reticulum were unable to detect cAMP generated by β2-adrenergic receptors in the T tubules [46], even though these organelles are juxtaposed a few hundred nanometres apart from each other at the dyadic junction of a cardiomyocyte [106]. Recent data suggests that there are differential responses in cAMP signalling even between the troponin complex and myosin binding protein C [12], which are located only a few nanometres apart from each other within the sarcomere [107]. 

FRET-based sensors for cAMP and downstream PKA activity have evolved from a highly demanding technique into a universally applicable tool over the last two decades. In the future, fine tuning this approach by targeting sensors to additional compartments, adjusting their sensitivity accordingly, and improving their dynamic range through engineering the fluorophores in a FRET pair will undoubtedly continue to refine our understanding of cAMP-regulated cardiomyocyte compartments.

## References

[1] Salazar N.C., Chen J., Rockman H.A. (2007). Cardiac GPCRs: GPCR signaling in healthy and failing hearts. Biochim. Biophys. Acta Biomembr..

[2] Kaupp U.B., Seifert R. (2002). Cyclic nucleotide-gated ion channels. Physiol. Rev..

[3] Schmidt M., Dekker F.J., Maarsingh H. (2013). Exchange Protein Directly Activated by cAMP (epac): A Multidomain cAMP Mediator in the Regulation of Diverse Biological Functions. Pharmacol. Rev..

[4] Schindler R.F.R., Brand T. (2016). The Popeye domain containing protein family—A novel class of cAMP effectors with important functions in multiple tissues. Prog. Biophys. Mol. Biol..

[5] Taylor S.S., Kim C., Cheng C.Y., Brown S.H.J., Wu J., Kannan N. (2008). Signaling through cAMP and cAMP-dependent protein kinase: Diverse strategies for drug design. Biochim. Biophys. Acta Proteins Proteom..

[6] Buxton I.L.O., Brunton L.L. (1983). Compartments of cyclic AMP and protein kinase in mammalian cardiomyocytes. J. Biol. Chem..

[7] Wang H., Robinson H., Ke H. (2007). The Molecular Basis for Different Recognition of Substrates by Phosphodiesterase Families 4 and 10. J. Mol. Biol..

[8] Sunahara R.K., Tesmer J.J., Gilman A.G., Sprang S.R. (1997). Crystal structure of the adenylyl cyclase activator Gsα. Science.

[9] Richards M., Lomas O., Jalink K., Ford K.L., Vaughan-Jones R.D., Lefkimmiatis K., Swietach P. (2016). Intracellular tortuosity underlies slow cAMP diffusion in adult ventricular myocytes. Cardiovasc. Res..

[10] Gesellchen F., Stangherlin A., Surdo N., Terrin A., Zoccarato A., Zaccolo M. (2011). Measuring spatiotemporal dynamics of cyclic AMP signaling in real-time using FRET-based biosensors. Methods Mol. Biol..

[11] Sprenger J.U., Nikolaev V.O. (2013). Biophysical techniques for detection of cAMP and cGMP in living cells. Int. J. Mol. Sci..

[12] Surdo N.C., Berrera M., Koschinski A., Brescia M., Machado M.R., Carr C., Wright P., Gorelik J., Morotti S., Grandi E. (2017). FRET biosensor uncovers cAMP nano-domains at β-adrenergic targets that dictate precise tuning of cardiac contractility. Nat. Commun..

[13] Horton J.K., Martin R.C., Kalinka S., Cushing A., Kitcher J.P., O’Sullivan M.J., Baxendale P.M. (1992). Enzyme immunoassays for the estimation of adenosine 3′,5′ cyclic monophosphate and guanosine 3′,5′ cyclic monophosphate in biological fluids. J. Immunol. Methods.

[14] Gilman A.G. (1970). A protein binding assay for adenosine 3’:5’-cyclic monophosphate. Proc. Natl. Acad. Sci. USA.

[15] Pradelles P., Grassi J., Chabardes D., Guiso N. (1989). Enzyme Immunoassays of Adenosine Cyclic 3’,5’-Monophosphate and Guanosine Cyclic 3’,5’-Monophosphate Using Acetylcholinesterase. Anal. Chem..

[16] Steiner A.L., Kipnis D.M., Utiger R., Parker C. (1969). Radioimmunoassay for the measurement of adenosine 3’,5’-cyclic phosphate. Proc. Natl. Acad. Sci. USA.

[17] Boularan C., Gales C. (2015). Cardiac cAMP: Production, hydrolysis, modulation and detection. Front. Pharmacol..

[18] Rochais F., Vandecasteele G., Lefebvre F., Lugnier C., Lum H., Mazet J.L., Cooper D.M.F., Fischmeister R. (2004). Negative feedback exerted by cAMP-dependent protein kinase and cAMP phosphodiesterase on subsarcolemmal cAMP signals in intact cardiac myocytes: An in vivo study using adenovirus-mediated expression of CNG channels. J. Biol. Chem..

[19] Fagan K.A., Schaack J., Zweifach A., Cooper D.M.F. (2001). Adenovirus encoded cyclic nucleotide-gated channels: A new methodology for monitoring cAMP in living cells. FEBS Lett..

[20] Rich T.C., Fagan K.A., Tse T.E., Schaack J., Cooper D.M., Karpen J.W. (2001). A uniform extracellular stimulus triggers distinct cAMP signals in different compartments of a simple cell. Proc. Natl. Acad. Sci. USA.

[21] Jurevicius J., Fischmeister R. (1996). cAMP compartmentation is responsible for a local activation of cardiac Ca^2+^ channels by β-adrenergic agonists. Proc. Natl. Acad. Sci. USA.

[22] Rich T.C., Tse T.E., Rohan J.G., Schaack J., Karpen J.W. (2001). In vivo assessment of local phosphodiesterase activity using tailored cyclic nucleotide-gated channels as cAMP sensors. J. Gen. Physiol..

[23] Adams S.R., Harootunian A.T., Buechler Y.J., Taylor S.S., Tsien R.Y. (1991). Fluorescence ratio imaging of cyclic AMP in single cells. Nature.

[24] Goaillard J.M., Vincent P., Fischmeister R. (2001). Simultaneous measurements of intracellular cAMP and l-type Ca2+ current in single frog ventricular myocytes. J. Physiol..

[25] Zaccolo M., De Giorgi F., Cho C.Y., Feng L., Knapp T., Negulescu P.A., Taylor S.S., Tsien R.Y., Pozzan T. (2000). A genetically encoded, fluorescent indicator for cyclic AMP in living cells. Nat. Cell Biol..

[26] Zaccolo M., Pozzan T. (2002). Discrete Microdomains with High Concentration of cAMP in Stimulated Rat Neonatal Cardiac Myocytes. Science.

[27] Kokkonen K., Kass D.A. (2017). Nanodomain Regulation of Cardiac Cyclic Nucleotide Signaling by Phosphodiesterases. Annu. Rev. Pharmacol. Toxicol..

[28] Mongillo M., McSorley T., Evellin S., Sood A., Lissandron V., Terrin A., Huston E., Hannawacker A., Lohse M.J., Pozzan T. (2004). Fluorescence Resonance Energy Transfer Based Analysis of cAMP Dynamics in Live Neonatal Rat Cardiac Myocytes Reveals Distinct Functions of Compartmentalized Phosphodiesterases. Circ. Res..

[29] Lehnart S.E., Wehrens X.H.T., Reiken S., Warrier S., Belevych A.E., Harvey R.D., Richter W., Jin S.L.C., Conti M., Marks A.R. (2005). Phosphodiesterase 4D deficiency in the ryanodine-receptor complex promotes heart failure and arrhythmias. Cell.

[30] Warrier S., Belevych A.E., Ruse M., Eckert R.L., Zaccolo M., Pozzan T., Harvey R.D. (2005). Beta-adrenergic- and muscarinic receptor-induced changes in cAMP activity in adult cardiac myocytes detected with FRET-based biosensor. Am. J. Physiol. Cell Physiol..

[31] Terrin A., Di Benedetto G., Pertegato V., Cheung Y.F., Baillie G., Lynch M.J., Elvassore N., Prinz A., Herberg F.W., Houslay M.D. (2006). PGE1 stimulation of HEK293 cells generates multiple contiguous domains with different [cAMP]: Role of compartmentalized phosphodiesterases. J. Cell Biol..

[32] Nikolaev V.O., Bünemann M., Hein L., Hannawacker A., Lohse M.J. (2004). Novel single chain cAMP sensors for receptor-induced signal propagation. J. Biol. Chem..

[33] Nikolaev V.O., Bünemann M., Schmitteckert E., Lohse M.J., Engelhardt S. (2006). Cyclic AMP imaging in adult cardiac myocytes reveals far-reaching β1-adrenergic but locally confined β2-adrenergic receptor-mediated signaling. Circ. Res..

[34] Koschinski A., Zaccolo M. (2017). Activation of PKA in cell requires higher concentration of cAMP than in vitro: Implications for compartmentalization of cAMP signalling. Sci. Rep..

[35] Tsalkova T., Blumenthal D.K., Mei F.C., White M.A., Cheng X. (2009). Mechanism of Epac activation. Structural and functional analyses of Epac2 hinge mutants with constitutive and reduced activities. J. Biol. Chem..

[36] Consonni S.V., Gloerich M., Spanjaard E., Bos J.L. (2012). cAMP regulates DEP domain-mediated binding of the guanine nucleotide exchange factor Epac1 to phosphatidic acid at the plasma membrane. Proc. Natl. Acad. Sci. USA.

[37] DiPilato L.M., Cheng X., Zhang J. (2004). Fluorescent indicators of cAMP and Epac activation reveal differential dynamics of cAMP signaling within discrete subcellular compartments. Proc. Natl. Acad. Sci. USA.

[38] Ponsioen B., Zhao J., Riedl J., Zwartkruis F., van der Krogt G., Zaccolo M., Moolenaar W.H., Bos J.L., Jalink K. (2004). Detecting cAMP-induced Epac activation by fluorescence resonance energy transfer: Epac as a novel cAMP indicator. EMBO Rep..

[39] Violin J.D., DiPilato L.M., Yildirim N., Elston T.C., Zhang J., Lefkowitz R.J. (2008). β2-Adrenergic receptor signaling and desensitization elucidated by quantitative modeling of real time cAMP dynamics. J. Biol. Chem..

[40] DiPilato L.M., Zhang J. (2009). The role of membrane microdomains in shaping beta2-adrenergic receptor-mediated cAMP dynamics. Mol. Biosyst..

[41] Klarenbeek J., Goedhart J., Van Batenburg A., Groenewald D., Jalink K. (2015). Fourth-generation Epac-based FRET sensors for cAMP feature exceptional brightness, photostability and dynamic range: Characterization of dedicated sensors for FLIM, for ratiometry and with high affinity. PLoS ONE.

[42] Di Benedetto G., Zoccarato A., Lissandron V., Terrin A., Li X., Houslay M.D., Baillie G.S., Zaccolo M. (2008). Protein kinase A type I and type II define distinct intracellular signaling compartments. Circ. Res..

[43] Nikolaev V.O., Moshkov A., Lyon A.R., Miragoli M., Novak P., Paur H., Lohse M.J., Korchev Y.E., Harding S.E., Gorelik J. (2010). β2-adrenergic receptor redistribution in heart failure changes cAMP compartmentation. Science.

[44] Llopis J., McCaffery J.M., Miyawaki A., Farquhar M.G., Tsien R.Y. (1998). Measurement of cytosolic, mitochondrial, and Golgi pH in single living cells with green fluorescent proteins. Proc. Natl. Acad. Sci. USA.

[45] Di Benedetto G., Scalzotto E., Mongillo M., Pozzan T. (2013). Mitochondrial Ca^2+^ uptake induces cyclic AMP generation in the matrix and modulates organelle ATP levels. Cell Metab..

[46] Sprenger J.U., Perera R.K., Steinbrecher J.H., Lehnart S.E., Maier L.S., Hasenfuss G., Nikolaev V.O. (2015). In vivo model with targeted cAMP biosensor reveals changes in receptor-microdomain communication in cardiac disease. Nat. Commun..

[47] Herget S., Lohse M.J., Nikolaev V.O. (2008). Real-time monitoring of phosphodiesterase inhibition in intact cells. Cell. Signal..

[48] Packer M., Carver J.R., Rodeheffer R.J., Ivanhoe R.J., DiBianco R., Zeldis S.M., Hendrix G.H., Bommer W.J., Elkayam U., Kukin M.L. (1991). Effect of oral milrinone on mortality in severe chronic heart failure. N. Engl. J. Med..

[49] Harbeck M.C., Chepurny O., Nikolaev V.O., Lohse M.J., Holz G.G., Roe M.W. (2006). Simultaneous optical measurements of cytosolic Ca^2+^ and cAMP in single cells. Sci. Signal..

[50] Odaka H., Arai S., Inoue T., Kitaguchi T. (2014). Genetically-encoded yellow fluorescent cAMP indicator with an expanded dynamic range for dual-color imaging. PLoS ONE.

[51] Hackley C.R., Mazzoni E.O., Blau J. (2018). cAMPr: A single-wavelength fluorescent sensor for cyclic AMP. Sci. Signal..

[52] Beavo J.A., Brunton L.L. (2002). Cyclic nucleotide research—Still expanding after half a century. Nat. Rev. Mol. Cell Biol..

[53] Zhang J., Ma Y., Taylor S.S., Tsien R.Y. (2001). Genetically encoded reporters of protein kinase A activity reveal impact of substrate tethering. Proc. Natl. Acad. Sci. USA.

[54] Zhang J., Hupfeld C.J., Taylor S.S., Olefsky J.M., Tsien R.Y. (2005). Insulin disrupts β-adrenergic signalling to protein kinase a in adipocytes. Nature.

[55] Allen M.D., Zhang J. (2006). Subcellular dynamics of protein kinase A activity visualized by FRET-based reporters. Biochem. Biophys. Res. Commun..

[56] Depry C., Allen M.D., Zhang J. (2011). Visualization of PKA activity in plasma membrane microdomains. Mol. Biosyst..

[57] Koschinski A., Zaccolo M. (2015). A novel approach combining real-time imaging and the patch-clamp technique to calibrate FRET-based reporters for cAMP in their cellular microenvironment. cAMP Signaling: Methods and Protocols.

[58] Chang C.R., Blackstone C. (2007). Cyclic AMP-dependent protein kinase phosphorylation of Drp1 regulates its GTPase activity and mitochondrial morphology. J. Biol. Chem..

[59] Hartzell H.C., Glass D.B. (1984). Phosphorylation of purified cardiac muscle C-protein by purified cAMP-dependent and endogenous Ca^2+^-calmodulin-dependent protein kinases. J. Biol. Chem..

[60] Solaro R.J., Moir A.J.G., Perry S.V. (1976). Phosphorylation of troponin I and the inotropic effect of adrenaline in the perfused rabbit heart. Nature.

[61] Marx S.O., Reiken S., Hisamatsu Y., Jayaraman T., Burkhoff D., Rosemblit N., Marks A.R. (2000). PKA Phosphorylation Dissociates FKBP12.6 from the Calcium Release Channel (Ryanodine Receptor). Cell.

[62] Burgers P.P., van der Heyden M.A., Kok B., Heck A.J., Scholten A. (2015). A systematic evaluation of protein kinase A-A-kinase anchoring protein interaction motifs. Biochemistry.

[63] Welch E.J., Jones B.W., Scott J.D. (2010). Networking with AKAPs: Context-dependent regulation of anchored enzymes. Mol. Interv..

[64] Kinderman F.S., Kim C., von Daake S., Ma Y., Pham B.Q., Spraggon G., Xuong N.H., Jennings P.A., Taylor S.S. (2006). A Dynamic Mechanism for AKAP Binding to RII Isoforms of cAMP-Dependent Protein Kinase. Mol. Cell.

[65] Maurice D.H., Ke H., Ahmad F., Wang Y., Chung J., Manganiello V.C. (2014). Advances in targeting cyclic nucleotide phosphodiesterases. Nat. Rev. Drug Discov..

[66] Akazawa H., Komuro I. (2003). Roles of cardiac transcription factors in cardiac hypertrophy. Circ. Res..

[67] Zippin J.H., Chen Y., Nahirney P., Kamenetsky M., Wuttke M.S., Fischman D.A., Levin L.R., Buck J. (2003). Compartmentalization of bicarbonate-sensitive adenylyl cyclase in distinct signaling microdomains. FASEB J..

[68] Zippin J.H., Farrell J., Huron D., Kamenetsky M., Hess K.C., Fischman D.A., Levin L.R., Buck J. (2004). Bicarbonate-responsive “soluble” adenylyl cyclase defines a nuclear cAMP microdomain. J. Cell Biol..

[69] Agarwal S.R., Miyashiro K., Latt H., Ostrom R.S., Harvey R.D. (2017). Compartmentalized cAMP responses to prostaglandin EP _2_ receptor activation in human airway smooth muscle cells. Br. J. Pharmacol..

[70] Sample V., DiPilato L.M., Yang J.H., Ni Q., Saucerman J.J., Zhang J. (2012). Regulation of nuclear PKA revealed by spatiotemporal manipulation of cyclic AMP. Nat. Chem. Biol..

[71] Yang J.H., Polanowska-Grabowska R.K., Smith J.S., Shields C.W., Saucerman J.J. (2014). PKA catalytic subunit compartmentation regulates contractile and hypertrophic responses to β-adrenergic signaling. J. Mol. Cell. Cardiol..

[72] Saucerman J.J., Zhang J., Martin J.C., Peng L.X., Stenbit A.E., Tsien R.Y., McCulloch A.D. (2006). Systems analysis of PKA-mediated phosphorylation gradients in live cardiac myocytes. Proc. Natl. Acad. Sci. USA.

[73] Agarwal S.R., MacDougall D.A., Tyser R., Pugh S.D., Calaghan S.C., Harvey R.D. (2011). Effects of cholesterol depletion on compartmentalized cAMP responses in adult cardiac myocytes. J. Mol. Cell. Cardiol..

[74] Wright P.T., Nikolaev V.O., O’Hara T., Diakonov I., Bhargava A., Tokar S., Schobesberger S., Shevchuk A.I., Sikkel M.B., Wilkinson R. (2014). Caveolin-3 regulates compartmentation of cardiomyocyte beta2-adrenergic receptor-mediated cAMP signaling. J. Mol. Cell. Cardiol..

[75] Mika D., Richter W., Westenbroek R.E., Catterall W.A., Conti M. (2014). PDE4B mediates local feedback regulation of 1-adrenergic cAMP signaling in a sarcolemmal compartment of cardiac myocytes. J. Cell Sci..

[76] Doenst T., Nguyen T.D., Abel E.D. (2013). Cardiac metabolism in heart failure: Implications beyond atp production. Circ. Res..

[77] Neubauer S. (2007). The Failing Heart—An Engine Out of Fuel. N. Engl. J. Med..

[78] Rizzuto R., Simpson A.W.M., Brini M., Pozzan T. (1992). Rapid changes of mitochondrial Ca^2+^ revealed by specifically targeted recombinant aequorin. Nature.

[79] Rizzuto R., Brini M., Murgia M., Pozzan T. (1993). Microdomains with high Ca^2+^ close to IP3-sensitive channels that are sensed by neighboring mitochondria. Science.

[80] Rizzuto R. (1998). Close Contacts with the Endoplasmic Reticulum as Determinants of Mitochondrial Ca^2+^ Responses. Science.

[81] Jouaville L.S., Pinton P., Bastianutto C., Rutter G.A., Rizzuto R. (1999). Regulation of mitochondrial ATP synthesis by calcium: Evidence for a long-term metabolic priming. Proc. Natl. Acad. Sci. USA.

[82] Chen L., Gong Q., Stice J.P., Knowlton A.A. (2009). Mitochondrial OPA1, apoptosis, and heart failure. Cardiovasc. Res..

[83] Cribbs J.T., Strack S. (2007). Reversible phosphorylation of Drp1 by cyclic AMP-dependent protein kinase and calcineurin regulates mitochondrial fission and cell death. EMBO Rep..

[84] Ma Y., Taylor S.S. (2008). A molecular switch for targeting between endoplasmic reticulum (ER) and mitochondria: Conversion of a mitochondria-targeting element into an ER-targeting signal in DAKAP1. J. Biol. Chem..

[85] Lefkimmiatis K., Leronni D., Hofer A.M. (2013). The inner and outer compartments of mitochondria are sites of distinct cAMP/PKA signaling dynamics. J. Cell Biol..

[86] Monterisi S., Lobo M.J., Livie C., Castle J.C., Weinberger M., Baillie G., Surdo N.C., Musheshe N., Stangherlin A., Gottlieb E. (2017). PDE2A2 regulates mitochondria morphology and apoptotic cell death via local modulation of cAMP/PKA signalling. Elife.

[87] Moss R.L., Razumova M., Fitzsimons D.P. (2004). Myosin crossbridge activation of cardiac thin filaments: Implications for myocardial function in health and disease. Circ. Res..

[88] Wehrens X.H.T., Lehnart S.E., Reiken S., Vest J.A., Wronska A., Marks A.R. (2006). Ryanodine receptor/calcium release channel PKA phosphorylation: A critical mediator of heart failure progression. Proc. Natl. Acad. Sci. USA.

[89] Ceholski D.K., Trieber C.A., Holmes C.F.B., Young H.S. (2012). Lethal, hereditary mutants of phospholamban elude phosphorylation by protein kinase A. J. Biol. Chem..

[90] Piacentino V., Weber C.R., Chen X., Weisser-Thomas J., Margulies K.B., Bers D.M., Houser S.R. (2003). Cellular basis of abnormal calcium transients of failing human ventricular myocytes. Circ. Res..

[91] Barbagallo F., Xu B., Reddy G.R., West T., Wang Q., Fu Q., Li M., Shi Q., Ginsburg K.S., Ferrier W. (2016). Genetically Encoded Biosensors Reveal PKA Hyperphosphorylation on the Myofilaments in Rabbit Heart Failure. Circ. Res..

[92] Liu S., Zhang J., Xiang Y.K. (2011). FRET-based direct detection of dynamic protein kinase a activity on the sarcoplasmic reticulum in cardiomyocytes. Biochem. Biophys. Res. Commun..

[93] Yin X., Cuello F., Mayr U., Hao Z., Hornshaw M., Ehler E., Avkiran M., Mayr M. (2010). Proteomics analysis of the cardiac myofilament subproteome reveals dynamic alterations in phosphatase subunit distribution. Mol. Cell. Proteom..

[94] Kensler R.W., Craig R., Moss R.L. (2017). Phosphorylation of cardiac myosin binding protein C releases myosin heads from the surface of cardiac thick filaments. Proc. Natl. Acad. Sci. USA.

[95] Ray K.P., England P.J. (1976). Phosphorylation of the inhibitory subunit of troponin and its effect on the calcium dependence of cardiac myofibril adenosine triphosphatase. FEBS Lett..

[96] Scott J.D., Santana L.F. (2010). A-Kinase Anchoring Proteins: Getting to the heart of the matter. Circulation.

[97] Carr D.W., Hausken Z.E., Fraser I.D.C., Stofko-Hahn R.E., Scott J.D. (1992). Association of the type II cAMP-dependent protein kinase with a human thyroid RII-anchoring protein: Cloning and characterization of the RII-binding domain. J. Biol. Chem..

[98] Carr D.W., Stofko-Hahn R.E., Fraser I.D.C., Bishop S.M., Acott T.S., Brennan R.G., Scott J.D. (1991). Interaction of the regulatory subunit (RII) of cAMP-dependent protein kinase with RII-anchoring proteins occurs through an amphipathic helix binding motif. J. Biol. Chem..

[99] Zoccarato A., Surdo N.C., Aronsen J.M., Fields L.A., Mancuso L., Dodoni G., Stangherlin A., Livie C., Jiang H., Sin Y.Y. (2015). Cardiac Hypertrophy Is Inhibited by a Local Pool of cAMP Regulated by Phosphodiesterase 2. Circ. Res..

[100] Sheridan C.M., Heist E.K., Beals C.R., Crabtree G.R., Gardner P. (2002). Protein kinase A negatively modulates the nuclear accumulation of NF-ATc1 by priming for subsequent phosphorylation by glycogen synthase kinase-3. J. Biol. Chem..

[101] Molkentin J.D., Lu J.R., Antos C.L., Markham B., Richardson J., Robbins J., Grant S.R., Olson E.N. (1998). A calcineurin-dependent transcriptional pathway for cardiac hypertrophy. Cell.

[102] Stangherlin A., Gesellchen F., Zoccarato A., Terrin A., Fields L.A., Berrera M., Surdo N.C., Craig M.A., Smith G., Hamilton G. (2011). CGMP signals modulate camp levels in a compartment-specific manner to regulate catecholamine-dependent signaling in cardiac myocytes. Circ. Res..

[103] Mongillo M., Tocchetti C.G., Terrin A., Lissandron V., Cheung Y.F., Dostmann W.R., Pozzan T., Kass D.A., Paolocci N., Houslay M.D. (2006). Compartmentalized phosphodiesterase-2 activity blunts β-adrenergic cardiac inotropy via an NO/cGMP-dependent pathway. Circ. Res..

[104] Li X., Nooh M.M., Bahouth S.W. (2013). Role of AKAP79/150 protein in β1-adrenergic receptor trafficking and signaling in mammalian cells. J. Biol. Chem..

[105] Neary M.T., Ng K.-E., Ludtmann M.H.R., Hall A.R., Piotrowska I., Ong S.-B., Hausenloy D.J., Mohun T.J., Abramov A.Y., Breckenridge R.A. (2014). Hypoxia signaling controls postnatal changes in cardiac mitochondrial morphology and function. J. Mol. Cell. Cardiol..

[106] Hayashi T., Martone M.E., Yu Z., Thor A., Doi M., Holst M.J., Ellisman M.H., Hoshijima M. (2009). Three-dimensional electron microscopy reveals new details of membrane systems for Ca2+ signaling in the heart. J. Cell Sci..

[107] Luther P.K., Winkler H., Taylor K., Zoghbi M.E., Craig R., Padron R., Squire J.M., Liu J. (2011). Direct visualization of myosin-binding protein C bridging myosin and actin filaments in intact muscle. Proc. Natl. Acad. Sci. USA.

